# Editorial Announcement

**Published:** 1915-05

**Authors:** 


### Editorial Announcement.

The Commission on Cancer of the Medical Society of the State of Pennsylvania announces that 50 medical journals have promised to devote space in the July issue to this topic. This journal is included in the list—with this proviso, that matter shall be submitted for publication that is worth publishing. One good paper has been promised. We would, of course, be pleased to receive conclusive evidence of the discovery of the cancer germ, or equally conclusive demonstration that there is no germ and that the disease is due to a particular metabolic process, or a report on a new and reliable specific biologic reaction. We shall, however, be disposed to accept papers depending upon original historic or archaeologic research or serious studies of literature, statistics, critical articles on diagnostic reactions, reports of interesting cases, in short, just such articles as may fall short of brilliant discovery but are valuable from the scientific standpoint. We do not believe in stirring up the subject merely for the sake of doing so. And we do not believe in a campaign until we know what we are fighting for and what we expect to accomplish.

---

**Haemophilia Treated With Horse Serum.** Henry C. Dozier, Ocala, Fla., Int. Jour. of Surg., Mch. 1915. Nothing known of grandparents. Parents and 2 sisters not bleeders. One brother living and two brothers dead of haemorrhage were also bleeders. Patient gives typic history of haemophilia and present attack consisted in haematuria. 10 days' treatment with gallic acid, calcium chlorid, tr. ferri chlor., adrenalin, ergot, etc., unavailing. Then, 10 c.c. of coagulose was administered hypodermatically every three hours, for about 3 1/2 days, the urine becoming free from blood after 24 hours and remaining so till about the same time after the last dose, when haemorrhage recurred. The stock of coagulose ran short and a new supply was not obtained till haemorrhage had recurred. Only 6 doses were then available. On this account, horse serum was administered, instead, 20 c.c. a day for three days and haemorrhage stopped, apparently permanently, so far as this attack was concerned, after three days.

